# Use of Pinching Nose Maneuver in a Patient With Severe Dysphagia Caused by Pseudobulbar Palsy

**DOI:** 10.7759/cureus.56116

**Published:** 2024-03-13

**Authors:** Kenjiro Kunieda, Yuki Natsume, Keishi Okamoto, Tomohisa Ohno, Ichiro Fujishima

**Affiliations:** 1 Neurology, Gifu University Graduate School of Medicine, Gifu, JPN; 2 Rehabilitation, Hamamatsu City Rehabilitation Hospital, Hamamatsu, JPN; 3 Dentistry, Hamamatsu City Rehabilitation Hospital, Hamamatsu, JPN; 4 Rehabilitation Medicine, Hamamatsu City Rehabilitation Hospital, Hamamatsu, JPN

**Keywords:** central pattern generator, pseudobulbar palsy, videofluorographic swallowing study, swallowing rehabilitation, swallowing impairment, cognitive impairment, oral phase, post-stroke dysphagia, stroke

## Abstract

Swallowing disorders resulting from pseudobulbar palsy are characterized by deficiencies in the oral preparatory and oral stages of the swallowing process. In certain cases, obstruction can occur when the tongue base comes into contact with the palate, impeding the intraoral bolus flow into the pharyngeal cavity. In this report, we discuss a case of severe pseudobulbar palsy, in which an intraoral bolus flowed into the pharyngeal cavity with pinching the nose. A 78-year-old man with a history of recurrent cerebral infarction was evaluated. The patient had severe dysphagia and cognitive impairment due to pseudobulbar palsy. A videofluoroscopic examination of swallowing (VF) was conducted while the patient was in a reclined position. In the oral cavity, when the bolus reached the posterior tongue section, the flow was hindered by the functional obstruction caused by the tongue base pressing against the palate. Despite the clinician’s instructions to swallow, the patient was unable to comply due to the severity of his cognitive impairment. To alleviate this obstruction, the clinician pinched the patient’s nose. This action opened the fauces, facilitating breathing and relieving the functional obstruction. Subsequently, the bolus flowed into the pharyngeal cavity and successfully flowed into the esophagus while swallowing. This maneuver was named the “pinching nose maneuver” (PNM). The PNM, as described here, can serve as a technique to improve the movement of an intraoral bolus into the pharyngeal cavity in patients with cognitive dysfunction.

## Introduction

Swallowing comprises four distinct stages: the oral preparatory, oral, pharyngeal, and esophageal. During the oral stage, contact of the tongue to the hard palate facilitates the bolus passage into the pharyngeal cavity. Dysphagia resulting from pseudobulbar palsy manifests as an impairment of the oral stage of swallowing [1-3]. Pseudobulbar palsy arises from bilateral corticobulbar tract dysfunction. In a few patients, the intraoral bolus becomes functionally obstructed due to the interaction between the palate and the tongue, hindering the bolus from flowing into the pharyngeal cavity. The central pattern generator (CPG) in the medulla governs the swallowing process of the pharyngeal stage [1]. The CPG remains unaffected in individuals with pseudobulbar palsy, thereby preserving pharyngeal swallowing function [1-3]. Consequently, ensuring a smooth flow of the intraoral bolus into the pharyngeal cavity becomes crucial for effective dysphagia rehabilitation in these patients. This report describes the case of a patient afflicted with dysphagia caused by pseudobulbar palsy, who successfully transported an intraoral bolus into the pharyngeal cavity with nasal pinching.

## Case presentation

The Patient

**Figure 1:**
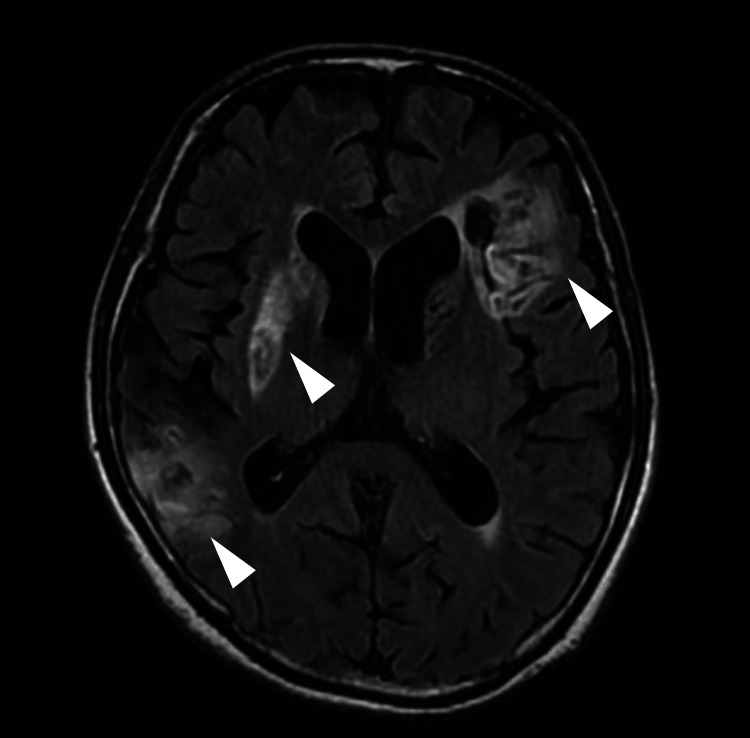
MRI-Fluid-Attenuated Inversion Recovery (FLAIR) images of the head showing cerebral infarction of the bilateral cerebral hemisphere The patient was diagnosed with a right hemispheric cerebral infarction due to occlusion of the right middle cerebral artery. His medical history included a left cerebral infarction. The patient presented with pseudobulbar palsy due to the bilateral cerebral hemispheric lesions (arrowheads).

A 78-year-old male developed a disturbance of consciousness and left-sided hemiplegia. His medical history included a left cerebral infarction three months prior. He exhibited mild right-sided hemiplegia but maintained ambulation and oral intake. Cognitive function was preserved, and he could communicate with his family. Upon initial evaluation, the Glasgow Coma Scale (GCS) indicated a level of consciousness at E4V1M5, and left-sided hemiplegia was observed. MRI-Fluid-Attenuated Inversion Recovery (FLAIR) images of the head showed cerebral infarction of the bilateral cerebral hemispheres resulting from occlusion of the right middle cerebral artery (Figure 1). The patient was diagnosed with recurrent hemispheric cerebral infarction. The etiology was a cardiogenic cerebral embolism. The patient underwent an emergency thrombectomy, and oral anticoagulation therapy was initiated. The patient presented with pseudobulbar palsy, severe dysphagia, and cognitive impairment due to the bilateral cerebral hemispheric lesions. He was provided nutrition through a feeding tube. The modified Rankin Scale (mRS) deteriorated from 2 to 5 compared to the period before the recurrent cerebral infarction. Shortly after the onset of symptoms, he displayed a pronounced difficulty in swallowing (i.e., severe dysphagia), which was assessed as level 2 on the Food Intake LEVEL Scale (FILS). This observer rating scale, consisting of 10 points, is utilized to quantify the severity of dysphagia. As a result of this condition, the individual underwent swallowing training without consuming food [4].

After 49 days since the onset of the disease, the patient was subsequently transferred to the hospital’s rehabilitation department. Despite cognitive impairment and difficulty following instructions, he conveyed his willingness by nodding in response to the questions and demonstrated his willingness to eat. The evaluation of cranial nerve palsy was constrained by severe cognitive impairment. The severity assessment of left hemiparesis using the Brunnstrom stage revealed a score of 3 for the upper extremity, 2 for the fingers, and 4 for the lower extremity.

**Figure 2:**
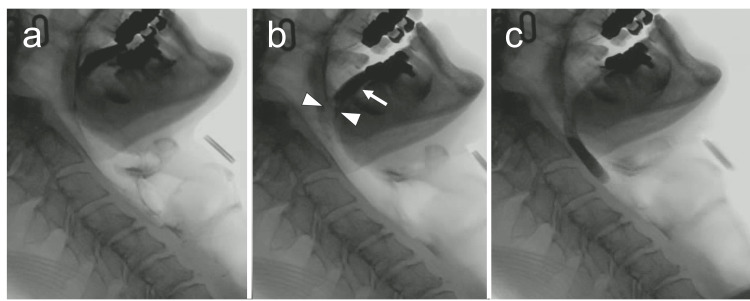
The process of pinching nose maneuver (PNM) a) Obstruction in the fauces resulting from the posterior tongue contacting the palate; b) Performing the “pinching nose maneuver.” When the examiner pinched the patient’s nose, the base of the tongue (indicated by the arrow) moved forward and downward, and the fauces opened (indicated by the arrowheads); c) Immediately after that, the bolus passed into the pharynx.

The patient required wheelchair use and showed a willingness to ingest orally. On day 57 after the onset of the stroke, the level of consciousness was E3V1M5 on the GCS score. At times, he could keep his eyes open for prolonged periods of time. He could respond to specific requests by nodding. Although some of his teeth were missing, he did not use dentures. An initial videofluoroscopic examination of swallowing (VF) revealed functional blockage, where the posterior section of the tongue was observed exerting pressure against the palate. Although we asked him to swallow, he could not follow the instructions, probably because of severe cognitive impairment. Swallowing rehabilitation techniques, such as adjusting the reclining posture, changing the bolus, ice massage, and K-point stimulation, were ineffective [5-8]. Thus, the intraoral bolus did not flow into the pharyngeal cavity (Figure 2).

The Pinching Nose Maneuver

The clinician pinched the nose with a hand when the bolus reached the tongue in the reclining position (Video 1). The patient opened his mouth and the inlet of the pharynx to breathe. The act of employing the pinching nose maneuver (PNM) removed the bolus blockage and enhanced the intraoral bolus flow into the pharyngeal cavity. Downward movement of the posterior tongue and elevation of the soft palate were observed. The oral cavity and pharynx were communicating, and the blockage was released. Shortly after that, the bolus moved into the pharynx, and the bolus passed through the pharyngeal cavity into the esophagus while swallowing reflex (Figure 2).

**Video 1:** The pinching nose maneuver (PNM) The contact between the base of the tongue and the palate blocked the bolus flow into the pharynx. Despite the clinician’s instructions to swallow, the patient was unable to deliver the intraoral bolus to the pharynx. When the clinician pinched the nose with a hand, the base of the tongue moved, establishing communication between the oral cavity and pharynx. Immediately after that, the bolus flowed into the pharynx. The reclining angle was 30 degrees.

After VF, a speech-language pathologist used the PNM for swallowing rehabilitation. Elevating the soft palate with a palatal-lingual prosthesis with a flexible lift (f-PLP) combined with PNM improves bolus transport from the oral cavity to the pharynx. His swallowing function improved to FILS level 4 (easy-to-swallow food less than the quantity of a meal (enjoyment level) was ingested orally).

## Discussion

This is, to our knowledge, the first report elucidating that pinching the nose could enhance the effective movement of an intraoral bolus into the pharyngeal cavity in a patient experiencing dysphagia caused by pseudobulbar palsy. If a patient's nose is pinched to breathe through the mouth, the base of the tongue detaches from the palate. Consequently, the functional blockage is released by the opening of the fauces, and the bolus flows into the pharyngeal cavity. The PNM can be performed easily in clinical situations because the clinician only pinches the patient’s nose.

The swallowing issue in this present case was a functional block caused by the continuous contact of the tongue with the palate. Furthermore, because patients commonly have cognitive impairment, it is often difficult to release functional blocks through instructions. Several methods exist for separating the tongue from the palate. f-PLP improves the intraoral bolus transportation into the pharynx [9,10]. It relieves the block in the oropharyngeal isthmus by elevating the soft palate. However, the PNM does not require specific tools or preparation. The "ee" maneuver could be used to release functional blockage by instructing the patient to pronounce "ee" [11]. This phenomenon is observed due to the articulatory movement of the base of the tongue towards the front and downward direction, coupled with the elevation of the soft palate during the pronunciation of the "ee” sound. However, patients who cannot follow commands, as in this case, cannot pronounce "ee.” In this case, the clinicians confirmed that if the PNM could release the functional block, the bolus could flow into the pharynx and be swallowed.

In this case, the pathology of the dysphagia was pseudobulbar palsy with cognitive dysfunction. The mechanism underlying this blockade remains unclear; however, swallowing apraxia may be involved. The CPG located in the nucleus tractus solitarius was not impaired, and the pharyngeal swallowing function was preserved [12]. Therefore, if the space between the oral cavity and the pharynx is bypassed and the bolus flows into the pharynx, the patient can swallow the bolus. Combined with the reclining posture, gravity facilitates the bolus flow into the pharynx. A reclining position of 30 degrees is recommended to facilitate bolus transport from the oral cavity to the pharynx. Additionally, a reclined position could be useful in reducing the risk of aspiration [13]. The PNM can be employed as a swallowing technique to enhance the movement of the bolus from the oral cavity to the pharynx in individuals suffering from cognitive impairment. The PNM may be a good indication for patients who are still unable to move the bolus from the oral cavity to the pharynx despite attempts at swallowing techniques (e.g., reclining position, ice massage, "ee" maneuvers). If the PNM is used to confirm that the pharyngeal swallowing function is preserved, swallowing rehabilitation may be facilitated using devices such as f-PLP. In this case, pseudobulbar palsy caused the pathophysiology of dysphagia. The problem in this case is not the velopharyngeal insufficiency. The use of f-PLP might help release functional contact between the tongue and soft palate, thereby opening the faucial isthmus. Continuous PNM could be a burden for caregivers. To reduce the burden and ensure consistent effectiveness, the creation of f-PLP, as demonstrated in this case, may be considered as in this case. Further research is needed to clarify whether PNM can adapt to other causes of swallowing disorders.

VF should be performed to ascertain whether the PNM is capable of effectively adjusting to alternative etiologies of dysphagia. The clinician confirmed the functional blockage and use of the PNM in the lateral view of the VF. Following the confirmation of the efficacy and safety of PNM with VF, it should be introduced in a clinical setting. Clinicians should be cautious regarding aspiration. When performing PNM in swallowing rehabilitation, clinicians should evaluate the level of consciousness and respiratory status, including factors such as respiratory rate. The clinical evaluation, such as choking and wet-hoarseness caused by pharyngeal residues, should also be evaluated. The use of oxygen saturation monitoring is recommended. Clinicians should ensure that the patients do not feel uncomfortable when their noses are pinched. The PNM is not necessarily applied to every swallowing throughout eating training or the meal. It may be discontinued if the patient can ingest orally without requiring the PNM. Even in situations where the PNM proves ineffective, clinicians should consider discontinuing it. The duration of performing the PNM should be regularly reassessed based on the clinical assessment of feeding status and willingness for oral intake. The PNM is not a standard swallowing maneuver, but a crucial maneuver to initiate swallowing, serving as a reminder for the patient to swallow. Further research is required to determine its effectiveness.

## Conclusions

The PNM can be an effective technique for enhancing the movement of the bolus from the oral cavity toward the pharynx in individuals with functional obstruction caused by contact between the tongue and palate. The PNM can be performed easily in clinical situations because the clinician only pinches the patient’s nose. This maneuver may be a good indication for patients who have difficulty following swallowing instructions. Further studies are required to evaluate the effectiveness of the PNM.
